# Continuous low serum levels of advanced glycation end products and low risk of cardiovascular disease in patients with poorly controlled type 2 diabetes

**DOI:** 10.1186/s12933-023-01882-9

**Published:** 2023-06-23

**Authors:** Tomoka Nakamura, Tetsuro Tsujimoto, Kazuki Yasuda, Kohjiro Ueki, Hiroshi Kajio

**Affiliations:** 1 Department of Diabetes, Endocrinology, and Metabolism, Center Hospital, National Center for Global Health and Medicine, 1-21-1 Toyama, Shinjuku-ku, Tokyo, 162-8655 Japan; 2 Department of Diabetes and Endocrinology, Toranomon Hospital Kajigaya, Kawasaki, Japan; 3 Department of Diabetes, Endocrinology and Metabolism, Kyorin University School of Medicine, Tokyo, Japan; 4 Department of Molecular Diabetic Medicine, Diabetes Research Center, Research Institute, National Center for Global Health and Medicine, Tokyo, Japan

**Keywords:** Diabetes mellitus, Advanced glycation end products, Diabetes related complication

## Abstract

**Background:**

Type 2 diabetes is associated with an increased risk of developing cardiovascular events. Previous studies have reported that advanced glycation end products (AGEs) were related to cardiovascular events in type 2 diabetes. However, data on associations between long-term AGEs and cardiovascular events in type 2 diabetes are lacking. This study aimed to determine whether a long-time shift in the levels of serum AGEs is associated with cardiovascular events in patients with poorly controlled type 2 diabetes.

**Methods:**

Two-time serum methyl-glyoxal-hydroimidazoline (MG-H1) levels were measured in 138 patients with type 2 diabetes whose mean glycated hemoglobin level was 10.1%. We categorized patients whose serum MG-H1 levels were < 2.8 µg/mL at both times as the continuous low MG-H1 group. The primary endpoints of this study were combined cardiovascular events, which were defined as heart disease, peripheral arterial disease, stroke, and all-cause death. Hazard ratios (HRs) for combined cardiovascular events with 95% confidence intervals (CIs) were calculated using the Cox proportional hazard models to compare the outcomes between the continuous low MG-H1 group and others.

**Results:**

The continuous low MG-H1 group was associated with a significantly lower risk than others in combined cardiovascular events after adjusting for possible confounders (HR: 0.50; 95% CI, 0.28–0.87; P = 0.02). Furthermore, the same relationship was observed in patients without a history of cardiovascular events.

**Conclusions:**

Continuous low serum MG-H1 levels are associated with a low frequency of diabetes-related complications in patients with poorly controlled type 2 diabetes.

**Supplementary Information:**

The online version contains supplementary material available at 10.1186/s12933-023-01882-9.

## Background

The number of patients with type 2 diabetes is increasing and is estimated to continue to increase because of an aging society, changes in lifestyle, and increasing prevalence of obesity [1, 2]. Type 2 diabetes is associated with an increased risk of developing microvascular complications, cardiovascular events, and death [3, 4]. The etiology of cardiovascular events, including coronary heart disease, peripheral arterial disease (PAD), heart failure, and stroke, in type 2 diabetes is based on inflammation and endothelial dysfunction [5]. However, the detailed mechanisms of the progression of cardiovascular events in type 2 diabetes remain unclear. Moreover, there is little evidence regarding the association between intensive glycemic control and risk reduction of cardiovascular events in type 2 diabetes. Some patients with long-term poor glycemic control develop no cardiovascular events. Early assessment of cardiovascular events in type 2 diabetes is sometimes difficult because currently available biomarkers, including glycated hemoglobin, have limitations in evaluating long-term glycemic control and identifying cardiovascular events risk with sufficient accuracy [6].

Advanced glycation end products (AGEs) were described as one of the causes of the development and progression of diabetes-related complications [7]. AGEs are a complex group of oxidant compounds formed by the nonenzymatic glycation of proteins, lipids, and nucleic acids. AGEs accumulate organs with advancing age and poor glycemic control [6]. They are believed to modify extracellular and intracellular proteins (collagen, elastin, and laminin) and lipids (low-density and high-density lipoproteins), which can change signaling, promote atherosclerosis, and cause cellular dysfunction. Other mechanisms include AGEs stimulate oxidative stress, inflammation, and apoptosis via interaction with their specific receptor (the receptor for AGEs: RAGE), expressed endothelial cells, macrophages, and monocytes, which play a role in atherosclerosis development [8]. Previous studies have reported that high serum AGEs levels were related to atherosclerosis and cardiovascular events in type 2 diabetes [9–14]. However, most studies have investigated only baseline AGEs levels. Data on associations between long-term AGEs and cardiovascular events in type 2 diabetes are lacking. Recently, we reported a case of poorly controlled type 2 diabetes with no progression of diabetes-related complications. This case had continuously low serum Methyl-glyoxal-hydroimidazoline (MG-H1), one of the AGEs, levels for 10 years despite poor glycemic control over 15 years [15]. MG-H1 is created by reacting arginine residues with methylglyoxal (MG), which is the most reactive dicarbonyl compound [16]. MG-H1 was one of the most abundant AGEs. MG, which is a precursor for AGEs, was a predictor of intima-media thickening, increasing pulse wave velocity, and systolic blood pressure elevation in patients with type 2 diabetes [17]. MG-H1 also has been reported to associate with the development of diabetic microvascular complications [10] and endothelial dysfunction [18]. Past reports including our case report inspired us to test the hypothesis that a subgroup of patients with diabetes who continuously exhibit low serum MG-H1 levels may be at a relatively low risk of experiencing cardiovascular events despite poor glycemic control. This study aimed to investigate the subgroup of patients with type 2 diabetes who have continuously low serum MG-H1 levels despite poor glycemic control and determine whether maintaining such levels in these patients is associated with a lower risk for cardiovascular events.

## Methods

### Study population

This cohort study used the data from MISSON-DM-ENDO, which included all patients admitted to the Department of Diabetes, Endocrinology, and Metabolism, Center Hospital, National Center for Global Health and Medicine in Tokyo, Japan. These patients provided written informed consent for their participation in the database. In the database, blood samples were taken immediately following the day after hospitalization and serum were stored at − 80 ℃ for future studies. We selected patients with diabetes who were admitted to be treated for hyperglycemia or examined and treated for diabetes-related complications at least twice between September 2003 (starting period of data collection) and April 2020 (n = 160). We included patients with type 2 diabetes and excluded patients with type 1 diabetes (n = 17) and other diabetes (n = 3). Moreover, we excluded patients who were treated for malignant tumors (n = 1) or human immunodeficiency virus (n = 1). Of 160 patients, 138 were eligible to participate in the study. We defined the most previous hospitalization as admission 1 and the most recent hospitalization as admission 2 within the study period for each patient. The median interval between admissions 1 and 2 was 4.8 years. Their mean glycated hemoglobin level was 10.1%. This study was approved by the institutional review boards of the National Center for Global Health and Medicine.

### Data collection

Baseline data, including age, sex, weight, height, diabetes duration, diabetes complications, medications, and laboratory findings, were collected from medical records. Body mass index (BMI) was calculated as weight in kilograms divided by height in meters squared. Obesity was defined as a BMI of 25 kg/m^2^ or more according to the obesity criteria in Japan [19]. The estimated glomerular filtration rate (eGFR) was calculated using the following formula, as recommended by the Japanese Society of Nephrology: eGFR (mL/min/1.73 m^2^) = 194 × Cre^− 1.094^ × Age^− 0.287^ (× 0.739 if the patient is a female) [20]. Dyslipidemia was defined as a previous diagnosis of hyperlipidemia or statin use. Hypertension was defined as a previous diagnosis of hypertension or antihypertensive drug use. We collected information about how many patients took angiotensin-converting enzyme inhibitors, angiotensin receptor blockers, and statins because these drugs have been known to affect the AGEs levels in both serum and tissue regardless of not influencing oxidative stress [21]. The serum levels of MG-H1 were measured using enzyme-linked immunosorbent assay (ELISA) (OxiSelect MG Competitive ELISA Kit #STA-811; Cell Biolabs, Inc., San Diego, CA, USA) according to the manufacturer’s instructions. To estimate a long-term shift of serum MG-H1 levels, we measured two-time serum levels of each patient at the most previous hospitalization (admission 1) and the most recent hospitalization (admission 2) during the study period. ELISA samples were run in duplicate. The intra coefficient of variation was 5% and the inter coefficient of variation was 8% from at least 3 independent tests for ELISA Kit.

### Outcome measurements

The primary endpoints of this study were combined cardiovascular events, including heart disease, PAD, stroke, and all-cause death. Heart disease was defined as myocardial infarction, angina pectoris (not vasospastic angina), performed artery revascularization, and new diagnosis or hospitalization for the treatment of heart failure. PAD was defined as a diagnosis based on the ankle brachial index or radiological images and performed artery revascularization. Stroke was defined as ischemic stroke, intracerebral hemorrhage, subarachnoid hemorrhage, and transit ischemic attack. Follow-up continued until March 31, 2022.

### Statistical methods

Data were presented as numbers (%) and means with standard deviations (± SD). Continuous and categorical variables were compared using t-tests and χ^2^ tests, respectively. The median serum MG-H1 level of all participants throughout two hospitalizations was 2.8 µg/mL. We categorized patients whose serum MG-H1 levels were < 2.8 µg/mL at both admission 1 and admission 2 as the continuous low MG-H1 group.

Patient characteristics at admission 1 were compared between the continuous low MG-H1 group and others. Setting admission 1 as the baseline, we performed Cox proportional hazards regression, and hazard ratios (HRs) and 95% confidence intervals (CIs) for outcome events in the continuous low MG-H1 group were compared with those of others. The first event was considered in the analyses of events if a patient developed multiple events. Outcome events below 10 were excluded from the analyses because a small number of outcome events make it difficult to analyze. A univariate model and three multivariable models with different sets of potential confounding variables were fitted for the outcome. Model 1 included adjustments for the following potential confounders: age, sex, and current smoking. Model 2 included adjustments for the potential confounders of model 1 plus diabetes duration, obesity, hypertension, and dyslipidemia. Model 3 included adjustments for the potential confounders of models 1 and 2 plus glycated hemoglobin and eGFR.

Moreover, we performed some sensitivity analyses to minimize residual confounding. Based on our hypothesis, patients whose serum MG-H1 level remained high may already develop cardiovascular events; therefore, we verified whether the same trend was observed in patients without cardiovascular events. First, we analyzed the absence of patients who developed any cardiovascular events between admissions 1 and 2. Second, we analyzed patients without a history of cardiovascular events before admission 1. Third, setting admission 2 as the baseline, the HRs for combined cardiovascular events in the continuous low MG-H1 group were assessed with those of others.

Further analyses were performed by setting admission 1 as the baseline to test the HRs of combined cardiovascular events in the continuous low MG-H1 group compared with those of others according to the following subgroups: age (< 65 or ≥ 65 years), sex (male or female), obesity (BMI of < 25 or ≥ 25 kg/m^2^), glycated hemoglobin level (< 10% or ≥ 10%), comorbidity of hypertension, and comorbidity of dyslipidemia. We examined the interactions between the continuous low MG-H1 group and these subgroups to investigate any confounding factors. These analyses were adjusted for variables described in model 3.

We classified the continuous high MG-H1 group as consisting of patients whose serum MG-H1 levels were above 2.8 µg/mL at both admissions 1 and 2 and the middle MG-H1 group as consisting of patients whose serum MG-H1 levels were above 2.8 µg/mL at either admission 1 or 2. We compared the outcomes between the continuous low MG-H1 group and the continuous high MG-H1 group or middle MG-H1 group.

P values < 0.05 were considered statically significant for all tests. All analyses were performed using the software package STATA version 14.0 (StataCorp, College Station, TX, USA).

## Results

### Baseline characteristics

The patient characteristics are presented in Table 1. At admission 1, the mean age (± SD) was 61.3 ± 14.2 years, 58.7% were male patients, and the mean glycated hemoglobin level was 10.1% ± 2.4%. The number of patients with a history of cardiovascular events was 40 (29.0%).

**Table 1:** **Characteristics of the study population**

Characteristic	All (n = 138)	Continuous low MG-H1 (n = 59)	Others (n = 79)	P value
Age (years)	61.3 ± 14.2	62.1 ± 13.9	60.7 ± 14.5	0.57
Male sex	81 (58.7%)	36 (61.0%)	45 (57.0%)	0.63
BMI (kg/m^2^)*	26.5 ± 6.3	26.4 ± 6.4	26.6 ± 6.3	0.86
Diabetes duration (years)	13.2 ± 11.3	11.6 ± 11.8	14.4 ± 10.9	0.16
Current smoker	35 (25.4%)	14 (23.7%)	21 (26.6%)	0.70
Glycated hemoglobin (%)				
at admission 1	10.1 ± 2.4	10.2 ± 2.3	10.1 ± 2.5	0.90
at admission 2	9.6 ± 1.9	9.3 ± 1.7	9.7 ± 2.0	0.27
Treatment for diabetes				
Oral agents	104 (75.4%)	43 (72.9%)	61 (77.2%)	0.56
Insulin	37 (26.8%)	13 (22.0%)	24 (30.4%)	0.27
Hypertension	96 (69.6%)	38 (64.4%)	58 (73.4%)	0.26
Use of angiotensin-converting enzyme inhibitors or angiotensin receptor blockers	62 (44.9%)	23 (39.0%)	39 (49.4%)	0.23
Dyslipidemia	100 (72.5%)	40 (67.8%)	60 (79.6%)	0.29
Use of statin	56 (40.6%)	18 (30.5%)	38 (48.1%)	0.04
Albuminuria	55 (42.3%)	22 (41.5%)	33 (42.9%)	0.88
Estimated glomerular filtration rate (mL/min/1.73 mm^2^)^†^	77.2 ± 28.3	75.5 ± 20.7	78.4 ± 33.0	0.57
History of cardiovascular events ‡	40 (29.0%)	10 (17.0%)	30 (38.0%)	0.007
Heart disease	32 (23.2%)	10 (17.0%)	22 (27.9%)	0.13
Stroke	13 (9.4%)	1 (1.7%)	12 (15.2%)	0.007
Peripheral arterial disease	8 (5.8%)	1 (1.7%)	7 (8.9%)	0.08
Serum MG-H1 level § (µg/mL)				
at admission 1	3.5 ± 3.0	1.8 ± 0.6	4.8 ± 3.5	< 0.001
at admission 2	3.7 ± 2.7	1.8 ± 0.5	5.1 ± 2.8	< 0.001
Period between admissions 1 and 2	4.8 ± 3.5	6.1 ± 3.2	3.8 ± 3.3	< 0.001

Values are presented as means ± standard deviations or numbers (%). Differences are evaluated using Chi-square analyses (categorical variables) and two-sample t-tests (continuous variables)

P values are calculated by comparing the variables in the continuous low MG-H1 group with those in others

Data are at the time of admission 1 except where specifically noted

*Body mass index (BMI) is calculated as weight in kilograms divided by height in meters squared

^†^The estimated glomerular filtration rate (eGFR) is calculated using the following formula, as recommended by the Japanese Society of Nephrology: eGFR (mL/min/1.73 m^2^) = 194 × Cre^− 1.094^ × Age^− 0.287^ (× 0.739 if the patient is a female)

‡Cardiovascular events consisted of heart disease, stroke, and peripheral arterial disease

§ MG-H1: methylglyoxal-derived hydroimidazolone-1

We compared the characteristics between the continuous low MG-H1 group and others. Age, sex, BMI, diabetes duration, eGFR, and comorbidities such as hypertension and dyslipidemia were not significantly different between groups. Patients who took statin were significantly fewer in the continuous low MG-H1 group. Glycated hemoglobin levels showed no significant difference among such groups, both at admissions 1 and 2 (admission 1: 10.2% ± 2.3% vs. 10.1% ± 2.5%, P = 0.90; admission 2: 9.3% ± 1.7% vs. 9.7% ± 2.0%, P = 0.27). Supplemental Fig. 1 shows the mean glycated hemoglobin level for 10 years since admission 1. The mean glycated hemoglobin levels during the follow-up period also did not differ between the continuous low MG-H1 group and others. The timing of the first admission was poorly controlled in all cases, but it was thought that glycemic control improved to some extent after the hospitalization. Additionally, glycemic control worsened again at the second hospitalization, which was different for each patient, and the results of Supplemental Fig. 1 suggest that the mean glycated hemoglobin was maintained in an improved state compared to the first time during long term follow-up. For a history of cardiovascular events, the continuous low MG-H1 group was related to fewer history of combined cardiovascular events than others (P = 0.007). The period between admissions 1 and 2 in the continuous low MG-H1 group was 6.1 ± 3.2 years, which was significantly longer than others (3.8 ± 3.3 years) (P < 0.001).

### Association between the continuous low MG-H1 and outcomes

During an average follow-up of 9.9 ± 4.1 years and a maximum follow-up of 18.6 years, beginning at admission 1, 62 (44.9%) participants developed combined cardiovascular events.

**Fig. 1:**
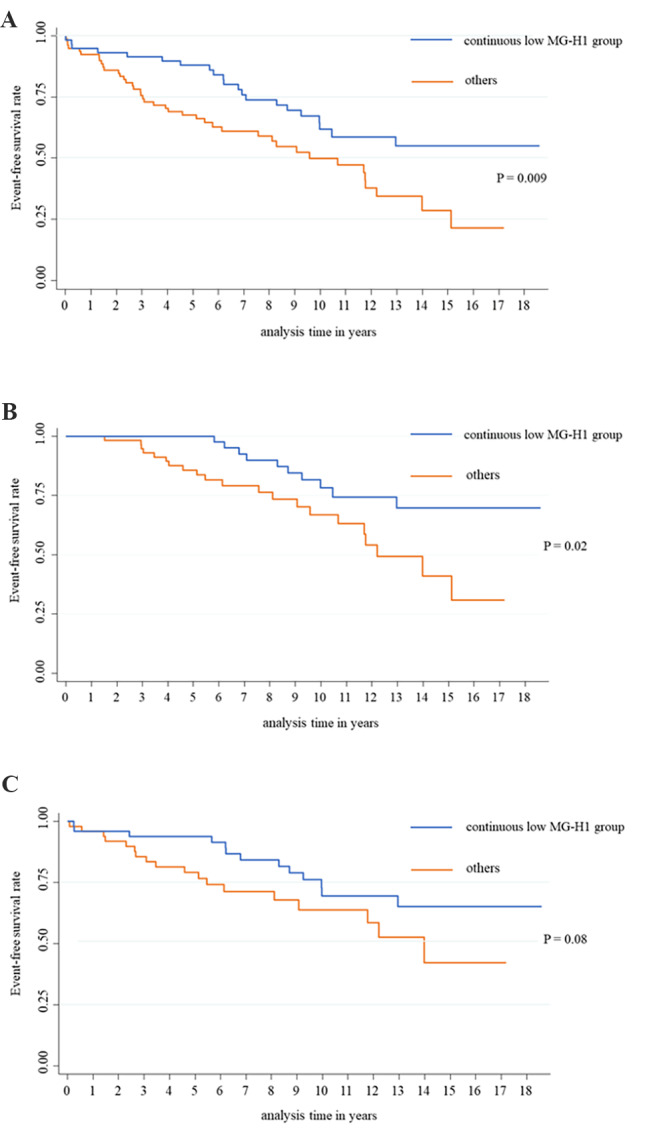
**Combined cardiovascular events according to serum MG-H1 levels. **Kaplan–Meier curve of combined cardiovascular events between the continuous low MG-H1 group and others (A). Kaplan–Meier curve of combined cardiovascular events between the continuous low MG-H1 group and others in patients without a history of cardiovascular events between admissions 1 and 2 (B). Kaplan–Meier curve of combined cardiovascular events between the continuous low MG-H1 group and others in patients without a history of cardiovascular events before admission 1 (C)

The Kaplan–Meier curve of combined cardiovascular events between the continuous low MG-H1 group and others is shown in Fig. 1A. The continuous low MG-H1 group was associated with a significantly lower risk than others in combined cardiovascular events (HR: 0.49, 95% CI: 0.29–0.83; P = 0.009). HRs for combined or each cardiovascular event in the continuous low MG-H1 group compared with others after adjusting for confounding variables are presented in Table 2. Significantly lower HRs in combined cardiovascular events in the continuous low MG-H1 group persisted even after adjusting for possible confounders (model 1: HR: 0.44, 95% CI: 0.26–0.76, P = 0.003; model 2: HR: 0.51, 95% CI: 0.29–0.88, P = 0.02; model 3: HR: 0.50, 95% CI: 0.28–0.87, P = 0.02). These results were almost unchanged even after changing a part of the adjustments of model 2 and 3 from “dyslipidemia” to “use of statins” (model 2: HR: 0.50, 95% CI: 0.29–0.88, P = 0.02; model 3: HR: 0.49, 95% CI: 0.28–0.86, P = 0.01). The continuous low MG-H1 group was associated with a significantly lower risk of all-cause death in models 1, 2, and 3. A similar trend was observed in heart disease and stroke. PAD was excluded from individual analyses because the number of cases was below 10.

**Table 2:** **Hazard ratios for cardiovascular events**

	All (n = 138)		Patients without any cardiovascular events between two admissions (n = 107)		Patients without a history of cardiovascular events before admission 1 (n = 98)	
	HR (95% CI)	P value	HR (95% CI)	P value	HR (95% CI)	P value
Combined cardiovascular events						
Unadjusted	**0.49 (0.29**–**0.83)**	**0.009**	**0.39 (0.18**–**0.84)**	**0.02**	0.53 (0.26–1.09)	0.08
Model 1	**0.44 (0.26**–**0.76)**	**0.003**	**0.34 (0.16**–**0.75)**	**0.01**	**0.43 (0.21**–**0.90)**	**0.03**
Model 2	**0.51 (0.29**–**0.88)**	**0.02**	**0.39 (0.18**–**0.88)**	**0.02**	**0.43 (0.20**–**0.94)**	**0.03**
Model 3	**0.50 (0.28**–**0.87)**	**0.02**	**0.37 (0.16**–**0.86)**	**0.02**	**0.41 (0.19**–**0.90)**	**0.03**
Heart disease						
Unadjusted	0.67 (0.36–1.23)	0.19	0.57 (0.25–1.32)	0.19	0.90 (0.36–2.21)	0.81
Model 1	0.65 (0.35–1.19)	0.16	0.54 (0.23–1.27)	0.16	0.85 (0.34–2.11)	0.85
Model 2	0.75 (0.40–1.41)	0.37	0.65 (0.27–1.56)	0.33	0.77 (0.29–1.99)	0.58
Model 3	0.76 (0.40–1.43)	0.39	0.64 (0.25–1.61)	0.34	0.76 (0.29–2.01)	0.59
Stroke						
Unadjusted	0.47 (0.18–1.21)	0.12	NA	NA	0.61 (0.17–2.15)	0.44
Model 1	0.41 (0.16–1.08)	0.07	NA	NA	0.41 (0.11–1.53)	0.19
Model 2	0.55 (0.20–1.53)	0.25	NA	NA	0.45 (0.11–1.82)	0.26
Model 3	0.54 (0.20–1.49)	0.24	NA	NA	0.31 (0.07–1.31)	0.11
Death						
Unadjusted	0.48 (0.20–1.19)	0.11	0.47 (0.16–1.40)	0.17	0.61 (0.16–2.28)	0.46
Model 1	**0.32 (0.12**–**0.84)**	**0.02**	0.32 (0.09–1.10)	0.07	0.36 (0.08–1.50)	0.16
Model 2	**0.35 (0.13**–**0.92)**	**0.03**	0.33 (0.09–1.17)	0.09	0.36 (0.08–1.64)	0.19
Model 3	**0.35 (0.13**–**0.93)**	**0.04**	0.31 (0.08–1.14)	0.08	0.27 (0.06–1.24)	0.09

NA = not applicable (the number of cases is below 10)

HRs = hazard ratios, CI = confidence interval

HRs and 95% CI for the outcomes in the continuous low MG-H1 group compared with those of others

Model 1 includes adjustments for the following potential confounders: age, sex, and current smoking

Model 2 includes adjustments for the potential confounders of model 1 plus dyslipidemia, hypertension, diabetes duration, and obesity

Model 3 includes adjustments for the potential confounders of models 1 and 2 plus glycated hemoglobin and eGFR.

### Sensitivity analyses

Except for patients who developed either cardiovascular event between admissions 1 and 2, a consistently low incidence of combined cardiovascular events persisted in the continuous low MG-H1 group (Table 2; Fig. 1B). Similar associations were observed for heart disease and death. In patients without a history of cardiovascular events before admission 1, the continuous low MG-H1 group was also associated with a low incidence of combined cardiovascular events (Table 2; Fig. 1C). Age, BMI, glycated hemoglobin level, eGFR, and comorbidities such as hypertension and dyslipidemia were also not significantly different between the continuous low MG-H1 group and others (Supplemental Table 1).

Additionally, setting admission 2 as the baseline, we investigated the HRs for combined cardiovascular events between the continuous low MG-H1 group and others. At 4.9 ± 3.2 years of follow-up, 50 (36.2%) patients developed combined cardiovascular events. Similarly, the continuous low MG-H1 group was associated with a significantly low incidence of combined cardiovascular events compared with others in any models (Supplemental Fig. 2, Supplemental Table 2). A similar relationship was observed in patients without a history of cardiovascular events before admission 2.

### Subgroup analyses

**Fig. 2:**
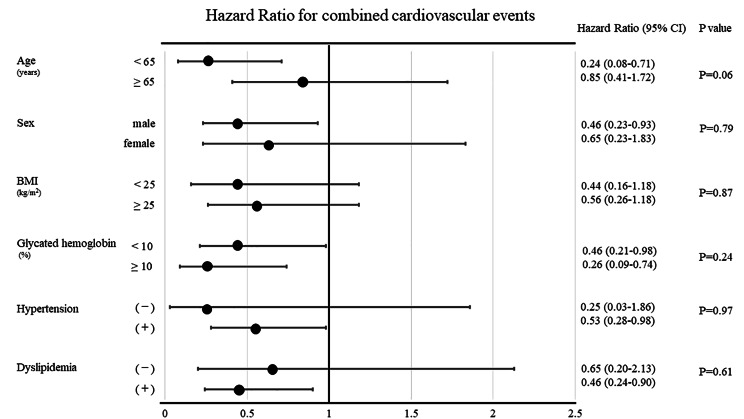
**Hazard ratio for combined cardiovascular events.** Hazard ratios for combined cardiovascular events between the continuous low MG-H1 group and others in different subgroups adjusted for variables described in model 3. Model 3 includes adjustments for the following potential confounders: age, sex, current smoking, dyslipidemia, hypertension, diabetes duration, obesity, glycated hemoglobin, and estimated glomerular filtration rate

The HRs for combined cardiovascular events in different subgroups adjusted for variables described in model 3 are presented in Fig. 2. Lower trends to combined cardiovascular events of the continuous low MG-H1 group were observed for any subgroup following multivariable adjustment. No significant interactions were observed between the MG-H1 group and subgroups of age, sex, BMI, glycated hemoglobin level, comorbidity of hypertension, and comorbidity of dyslipidemia.

### Continuous low MG-H1 group versus continuous high MG-H1 group or middle MG-H1 group

We compared the outcomes between the continuous low MG-H1 group (n = 59) and continuous high MG-H1 group (n = 58) or middle MG-H1 group (n = 21). The continuous low MG-H1 group was associated with a significantly lower risk than the continuous high MG-H1 group in combined cardiovascular events in any model (Supplemental Fig. 3A, Supplemental Table 3). The same relationship was observed in patients without a history of cardiovascular events (Supplemental Fig. 3B, 3 C). The continuous low MG-H1 group also had a lower risk of developing combined cardiovascular events than the middle MG-H1 group in a univariate model, although the difference was not significant (HR: 0.62, 95% CI: 0.28–1.36; P = 0.23).

## Discussion

This study demonstrated that some patients with type 2 diabetes has constantly low serum levels of AGEs despite having poor glycemic control for a long time, and these persistently low AGEs levels were associated with a low risk for combined cardiovascular events. The AGEs levels may be affected by age, habits, diabetes duration, renal clearance, drugs, and comorbidities [22]. In the present study, baseline characteristics such as age, BMI, eGFR, and comorbidities of hypertension and dyslipidemia were not significantly different between the continuous low MG-H1 group and others. After accounting for possible confounders, the low risk of combined cardiovascular events in the continuous low MG-H1 group was observed. Even if we excluded patients who developed any combined cardiovascular events between two hospitalizations or before admission 1, our findings were robust. The conclusions of the study were largely unchanged when different baselines were used in the analyses. No significant interactions were observed between the MG-H1 group and factors including age, sex, BMI, glycated hemoglobin level, and comorbidities such as hypertension and dyslipidemia.

Previous studies showed higher baseline serum MG-H1 levels associated with severe endothelial dysfunction, type 2 diabetes, and history and incidence of cardiovascular events [11, 18]. Monnier et al. reported that the association between baseline skin AGEs levels and carotid intima–media thickness was observed after a 6-year follow-up; however, it was no longer noted after 12 years [23]. Another study reported that a 4-week high-dietary AGEs intake changed the urine and plasma AGEs levels; however, no difference in macrovascular function assessed by flow-mediated dilation, inflammatory markers, or lipid profile was noted between the low- and high-dietary AGEs groups [24]. Therefore, a single measurement of AGEs levels may have limited significance for determining long-term diabetes-related complications. We reported a subgroup of patients with type 2 diabetes who maintained low serum AGEs levels, which have been described to accumulate with aging and hyperglycemia [6], despite their long-time poor glycemic control and longer measurement interval. This subgroup had a lower risk of developing combined cardiovascular events than the middle MG-H1 group, although the difference was not significant. Few studies have focused on continuous low MG-H1 levels in type 2 diabetes with long-time poor glycemic control. To our knowledge, this study is the first to confirm the association between long-term serum MG-H1 levels and cardiovascular events in patients with poorly controlled type 2 diabetes.

Previous studies showed that AGEs had a weak association with glycated hemoglobin levels [11, 14]. Our findings are consistent with those of previous findings. Glycated hemoglobin level was not significantly different between the continuous low MG-H1 group and others both at admissions 1 and 2, and was maintained at high levels in such groups for 10 years regrettably. Patients with poor metabolic control exist even if followed by specialists because they could not perform diet therapy and/or exercise therapy according to the recommendations of specialists, and/or specialists could not add therapeutic drugs due to patient refusal or side effects. Therapeutic inertia, which is a challenge to overcome in diabetes care, may be involved in some cases [25]. On the other hand, our data support that serum AGEs levels can be a key mediator of diabetes-related complications independently with glycated hemoglobin levels. In the clinical environment, continuous measurement of serum AGEs to patients with poorly controlled diabetes may be helpful to predict cardiovascular events.

Previous study results revealed that many factors other than hyperglycemia may regulate AGEs. Some drugs, such as statins, angiotensin-converting enzyme inhibitors, angiotensin receptor blockers, thiazolidinediones, and pyridoxamine, can affect AGEs concentration and RAGE expression [21]. AGEs are particularly found in high concentrations in foods rich in both protein and fat, mostly of animal origin, and eating such food items cooked at high and dry heat can elevate AGEs level [26]. Excessive dietary AGEs intake is associated with gut structure alterations, causing enhanced gut barrier dysfunction, enteric neuron expression changes, microbial dysbiosis, and inflammation [27]. Another significant source of exogenous AGEs is tobacco smoke exposure [28]. A previous study compared AGEs levels quantified using skin autofluorescence between children with type 1 diabetes and their siblings without diabetes and revealed a significant correlation in skin autofluorescence levels between siblings when adjusted for glycated hemoglobin levels and age [29]. Another study investigated serum AGEs levels in normal twins and revealed highly significant and higher twin correlations for AGEs in monozygotic than in dizygotic twins, distinct from fasting glucose and glycated hemoglobin [30]. Considering the above information, our patients with continuous low serum MG-H1 levels might have been affected by some drugs, oral AGEs intake, genetic factors, and/or environmental factors from the early stage of life other than hyperglycemia. A previous study suggested that MG, which is a precursor for MG-H1, primarily accumulates inside the vascular endothelial cells and increases oxidative stress, vascular resistance, insulin resistance, and salt sensitivity [17]. MG-H1 can be a major factor inducing cardiovascular events. The detailed mechanism of continued low MG-H1 remains unclear, but continued low cytotoxic and dangerous MG-H1 levels may be associated with a reduced risk of cardiovascular events. Further research is needed, including causal relationships.

This study had some potential limitations. First, it had a small sample size. This study was performed at a single center. Therefore, large-scale studies at multiple centers are necessary to extrapolate the results to a wide range of individuals. Previous report revealed that AGEs were strongly associated with PAD in patients with type 2 diabetes [31]. Our results were also consistent in that AGEs were associated with the occurrence of cardiovascular events. PAD was excluded from the individual analysis because of a small number of occurrences in our study, thus we need to evaluate PAD in large-scale studies.　Second, there may be an evaluation of serum AGEs levels. We chose ELISA to evaluate serum MG-H1 levels. There are several methods available to measure AGEs such as Liquid chromatography-tandem mass spectroscopy and skin autofluorescence [6, 23]. Liquid chromatography-tandem mass spectroscopy requires specialized equipment and highly trained skill, making it inappropriate for large samples. Skin autofluorescence is not specific to AGEs because of the overlap of spectra, and it is not easily available in daily medical practice. No evidence has revealed that any specific measure offers any advantages. Moreover, there are many AGEs, and true levels of each AGEs are unknown. A previous study, which compared serum MG-H1 levels between patients with newly diagnosed diabetes and those with established diabetes, reported that their serum MG-H1 levels were 4.00 ± 0.09 and 3.09 ± 0.13 µg/mL, respectively [18]. This report used the same ELISA kit. Taking this data into account, the cutoff value of the present study seems appropriate. Other AGEs, including Nε-carboxymethyl lysine or pentosidine, were not measured in our study, thus whether other AGEs can predict cardiovascular events is unclear. However, in vitro studies and animal experiments indicated MG, which is a precursor for AGEs, to play a major role in vascular damage to endothelial cells and the development of hypertension, insulin resistance, and nephropathy [32, 33]. Additionally, MG is produced not only by hyperglycemia but also by a variety of proteins and conditions [34]. Hence, MG-H1 may be a better candidate for cardiovascular events independently of glycemic control. We evaluated the serum MG-H1 levels only twice, thus more frequent evaluation may have been needed in the course of the long follow-up. Third, a causal relationship cannot be established from observational data. Continuous low serum AGEs levels may themselves cause differences in inflammatory or metabolic pathways and lead to a low frequency of cardiovascular events or death; conversely, individuals with a less likelihood of cardiovascular event development or death may be less likely to form and accumulate AGEs. Lastly, there may be confounding by unmeasured factors or ones that are difficult to quantify. For example, this study was unable to collect dietary data, including AGEs content, although our patients were all admitted and had fasted, and some conditions, such as the last meal, seemed to be similar. A prospective study is needed to collect information on dietary AGEs intake. As mentioned above, AGEs originate from either endogenous or exogenous sources. Some unmeasured or unknown factors may affect the continuously low levels of serum MG-H1.

In conclusion, this study found a subgroup of patients with type 2 diabetes whose serum MG-H1 levels were continuously low despite long-time poor glycemic control. In addition, maintaining low serum MG-H1 levels was associated with a low risk of developing cardiovascular events. Longitudinal studies with larger populations of AGEs as useful noninvasive biomarkers should be conducted to develop therapeutic strategies necessary for preventing diabetes-related complications.

## Electronic supplementary material

Below is the link to the electronic supplementary material.

Supplementary Material

## Data Availability

The datasets used and analyzed during the current study are available from the corresponding author on reasonable request.

## Abbreviations

MG-H1: methyl-glyoxal-hydroimidazoline
HRs: hazard ratios
CIs: confidence intervals
PAD: peripheral arterial disease
AGEs: advanced glycation end products
RAGE: the receptor for AGEs
BMI: body mass index
eGFR: estimated glomerular filtration rate
MG: methylglyoxal
ELISA: enzyme-linked immunosorbent assay
SD: standard deviations
