# Supplementary material for: Continuous low serum levels of advanced glycation end products and low risk of cardiovascular disease in patients with poorly controlled type 2 diabetes
Source: Cardiovasc Diabetol. 2023 Jun 23;22:147. doi: 10.1186/s12933-023-01882-9 (PMC10290294; doi:10.1186/s12933-023-01882-9)

Continuous low serum levels of advanced glycation end products and low risk of cardiovascular disease in patients with poorly controlled type 2 diabetes

Tomoka Nakamura, MD[a], Tetsuro Tsujimoto, MD, PhD[a,b]*, Kazuki Yasuda, MD, PhD[c], Kohjiro Ueki, MD, PhD[a,d], Hiroshi Kajio, MD, PhD[a]

a. Department of Diabetes, Endocrinology, and Metabolism, Center Hospital, National Center for Global Health and Medicine, Tokyo, Japan
b. Department of Diabetes and Endocrinology, Toranomon Hospital Kajigaya, Kawasaki, Japan.
c. Department of Diabetes, Endocrinology and Metabolism, Kyorin University School of Medicine, Tokyo, Japan
d. Department of Molecular Diabetic Medicine, Diabetes Research Center, Research Institute, National Center for Global Health and Medicine, Tokyo, Japan.

*Correspondence:
Tetsuro Tsujimoto, M.D., Ph.D.
Department of Diabetes, Endocrinology and Metabolism, Center Hospital, National Center for Global Health and Medicine, 1-21-1 Toyama, Shinjuku-ku, Tokyo 162-8655, Japan (e-mail: ttsujimoto@hosp.ncgm.go.jp).

Supplemental Table 1: **Characteristics of the study population (Patients without a history of cardiovascular events before admission 1)**

Characteristic	All (n = 98)	Continuous low MG-H1 (n = 49)	Others (n = 49)	P value
Age (years)	59.2 ± 15.4	60.9 ± 14.3	57.5 ± 16.4	0.28
Male sex	54 (55.1%)	29 (59.2%)	25 (51.0%)	0.42
BMI (kg/m^2)*	26.2 ± 6.9	26.1 ± 6.7	26.3 ± 7.2	0.89
Diabetes duration (years)	12.1 ± 11.4	10.4 ± 11.6	13.8 ± 11.1	0.15
Current smoker	29 (29.6%)	14 (28.6%)	15 (30.6%)	0.83
Glycated hemoglobin (%)				
at admission 1	10.3 ± 2.4	10.4 ± 2.4	10.2 ± 2.5	0.69
at admission 2	9.7 ± 2.0	9.5 ± 1.7	9.9 ± 2.2	0.32
Treatment for diabetes				
Oral agents	73 (74.5%)	35 (71.4%)	38 (77.6%)	0.49
Insulin	22 (22.5%)	9 (18.4%)	13 (26.5%)	0.33

Hypertension	59 (60.2%)	29 (59.2%)	30 (61.2%)	0.84
Use of angiotensin-converting enzyme inhibitors or angiotensin receptor blockers	34 (34.7%)	17 (34.7%)	17 (34.7%)	1.00
Dyslipidemia	70 (71.4%)	33 (67.4%)	37 (75.5%)	0.37
Use of statin	36 (36.7%)	13 (26.5%)	23 (46.9%)	0.04
Albuminuria	32 (34.4%)	15 (34.1%)	17 (34.7%)	0.95
Estimated glomerular filtration rate (mL/min/1.73 mm$^{2)\dagger}$	81.5 ± 29.5	77.4 ± 20.9	85.6 ± 35.9	0.17

Values are presented as means ± standard deviations or numbers (%). Differences were evaluated using Chi-square analyses for categorical variables and two-sample *t*-tests for continuous variables.
P values were calculated by comparing the variables in the continuously low MG-H1 group with those in others.
Data are at the time of admission 1 except where specifically noted.

*Body mass index (BMI) was calculated as weight in kilograms divided by height in meters squared.
†The estimated glomerular filtration rate (eGFR) was calculated using the following formula, as recommended by the Japanese Society of Nephrology: eGFR (mL/min/1.73 m^2) = 194 × Cre$^{-1.094}$ × Age$^{-0.287}$ (× 0.739 for female patients).

Supplemental Table 2: **Hazard ratios for cardiovascular events (setting admission 2 as the baseline)**

	All (n = 138)		Patients without a history of cardiovascular events before admission 2 (n = 79)	
	HR (95% CI)	P value	HR (95% CI)	P value
Unadjusted	**0.51 (0.27–0.98)**	**0.04**	0.73 (0.23–2.27)	0.58
Model 1	**0.46 (0.24–0.88)**	**0.02**	0.80 (0.24–2.67)	0.72
Model 2	**0.44 (0.23–0.84)**	**0.01**	0.60 (0.15–2.30)	0.45
Model 3	**0.47 (0.24–0.93)**	**0.03**	0.44 (0.11–1.87)	0.27

HRs = hazard ratios, CI = confidence interval
HRs and 95% CI for the outcomes in the continuous low MG-H1 group compared with those of others.
Model 1 includes adjustments for the following potential confounders: age, sex, and current smoking.
Model 2 includes adjustments for the potential confounders of model 1 plus dyslipidemia, hypertension, diabetes duration, and obesity.
Model 3 includes adjustments for the potential confounders of models 1 and 2 plus glycated hemoglobin and eGFR.

Supplemental Table 3: **Hazard ratios for cardiovascular events (continuous low MG-H1 group vs. continuous high MG-H1 group)**

	All (n = 117)		Patients without any cardiovascular events between two admissions (n = 90)		Patients without a history of cardiovascular events before admission 1 (n = 82)	
	HR (95% CI)	P value	HR (95% CI)	P value	HR (95% CI)	P value
Unadjusted	**0.45 (0.26–0.79)**	**0.01**	**0.37 (0.17–0.83)**	**0.02**	0.50 (0.23–1.10)	0.08
Model 1	**0.40 (0.23–0.71)**	**0.002**	**0.34 (0.15–0.78)**	**0.01**	**0.38 (0.17–0.85)**	**0.02**
Model 2	**0.47 (0.26–0.85)**	**0.01**	**0.36 (0.15–0.98)**	**0.02**	**0.38 (0.16–0.90)**	**0.03**
Model 3	**0.47 (0.26–0.86)**	**0.01**	**0.36 (0.15–0.88)**	**0.03**	**0.37 (0.15–0.89)**	**0.03**

HRs = hazard ratios, CI=confidence interval
HRs and 95% CI for the outcomes in the continuous low MG-H1 group compared with those of the continuous high MG-H1 group
Model 1 includes adjustments for the following potential confounders: age, sex, and current smoking.
Model 2 includes adjustments for the potential confounders of model 1 plus dyslipidemia, hypertension, diabetes duration, and obesity.
Model 3 includes adjustments for the potential confounders of models 1 and 2 plus glycated hemoglobin and eGFR.

Supplemental Figure 1: Transition of glycated hemoglobin levels

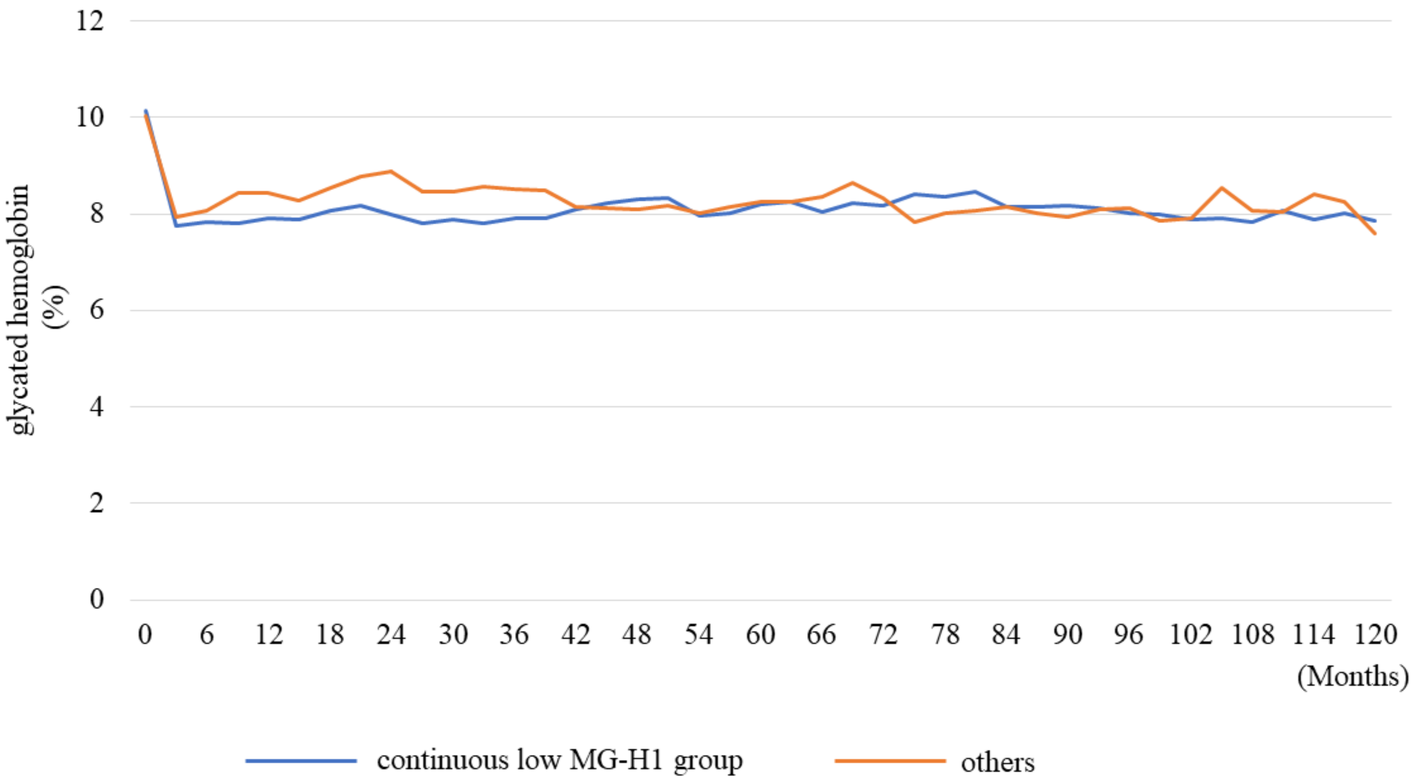

Supplemental Figure 2

Combined cardiovascular events according to serum MG-H1 levels (setting admission 2 as the baseline)

Kaplan–Meier curve of combined cardiovascular events between the continuous low MG-H1 group and others (setting admission 2 as the baseline).

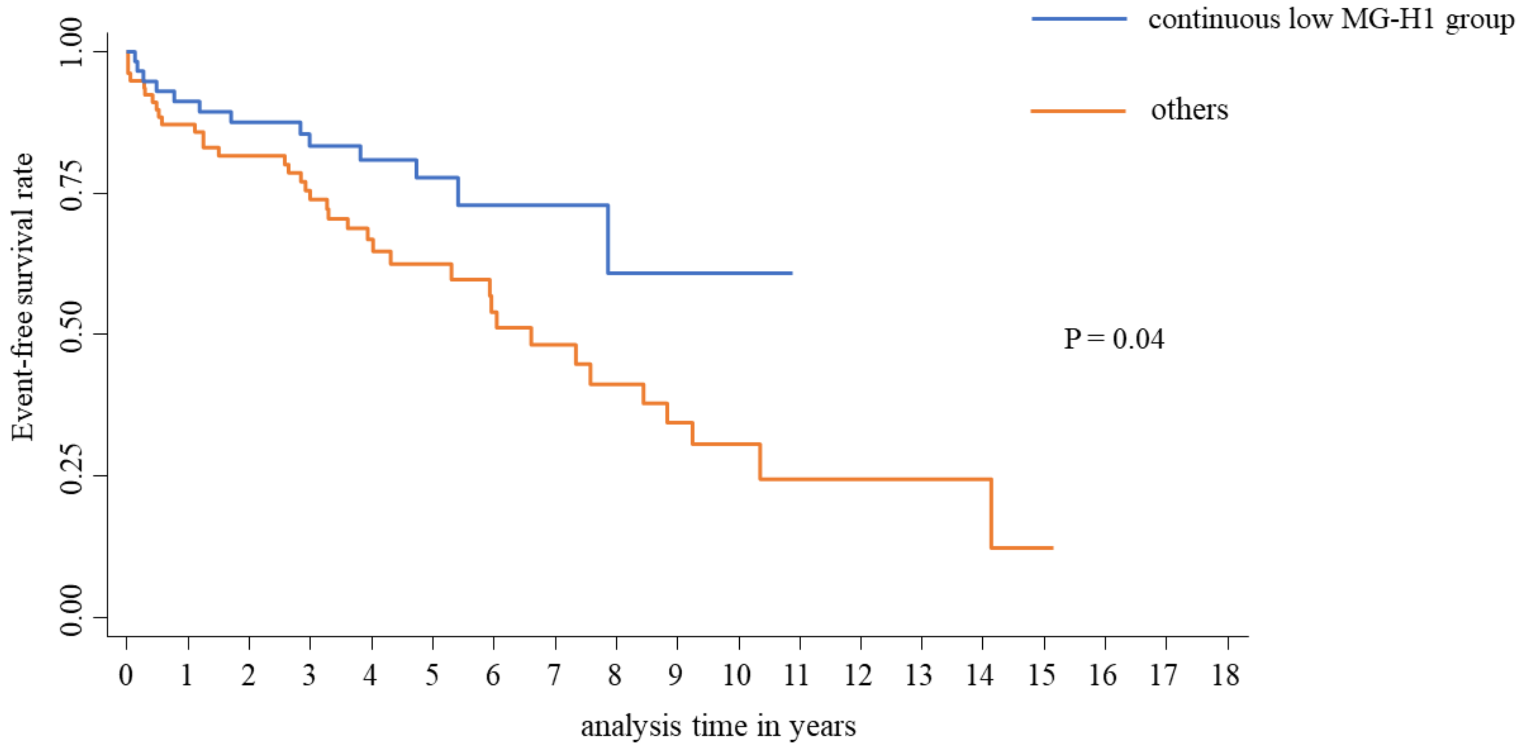

Supplemental Figure 3

Combined cardiovascular events according to serum MG-H1 levels

Kaplan–Meier curve of combined cardiovascular events between the continuous low MG-H1, middle MG-H1, and continuous high MG-H1 groups (A).

Kaplan–Meier curve of combined cardiovascular events between the continuous low MG-H1, middle MG-H1, and continuous high MG-H1 groups in patients without a history of cardiovascular events between admissions 1 and 2 (B).

Kaplan–Meier curve of combined cardiovascular events between the continuous low MG-H1, middle MG-H1, and continuous high MG-H1 groups in patients without a history of cardiovascular events before admission 1 (C).

Supplemental Figure 3A

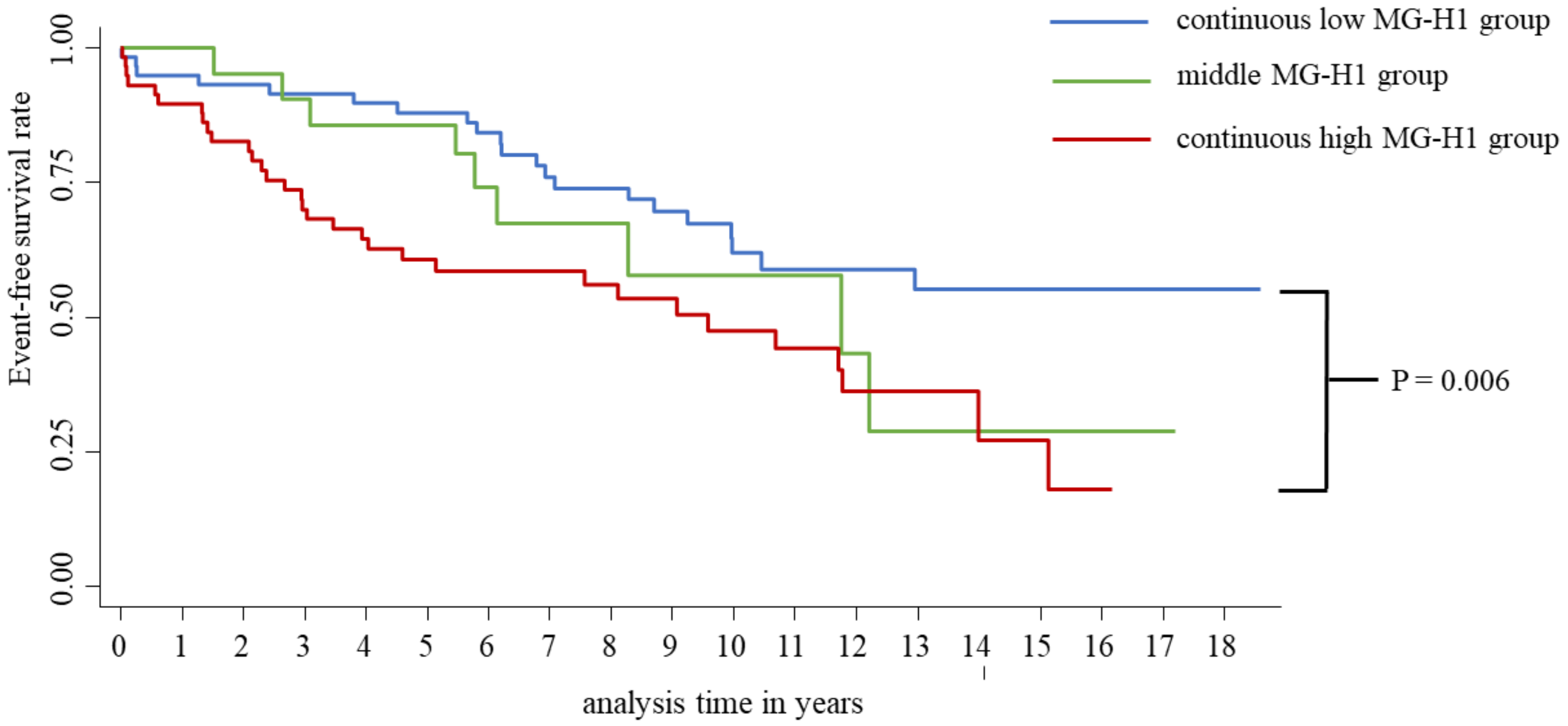

Supplemental Figure 3B

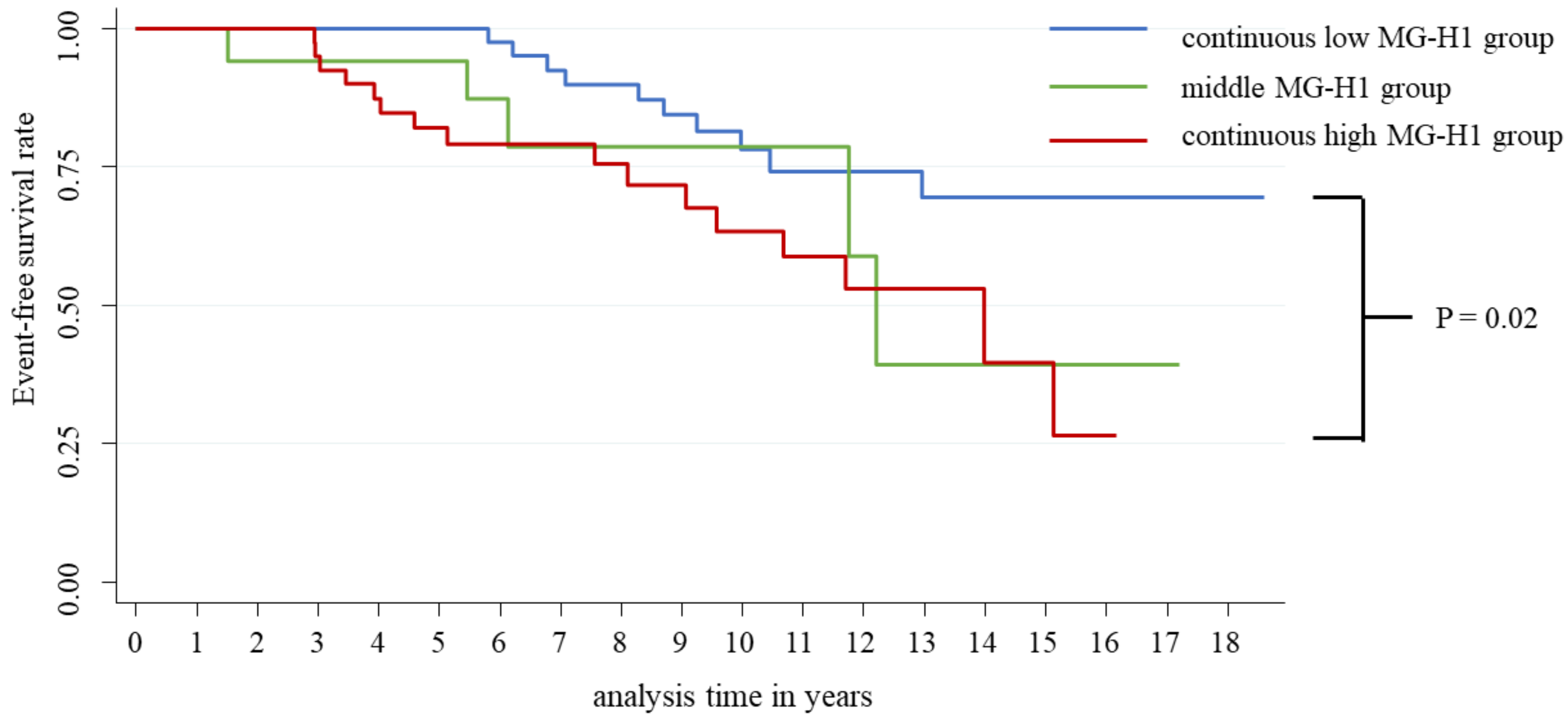

Supplemental Figure 3C

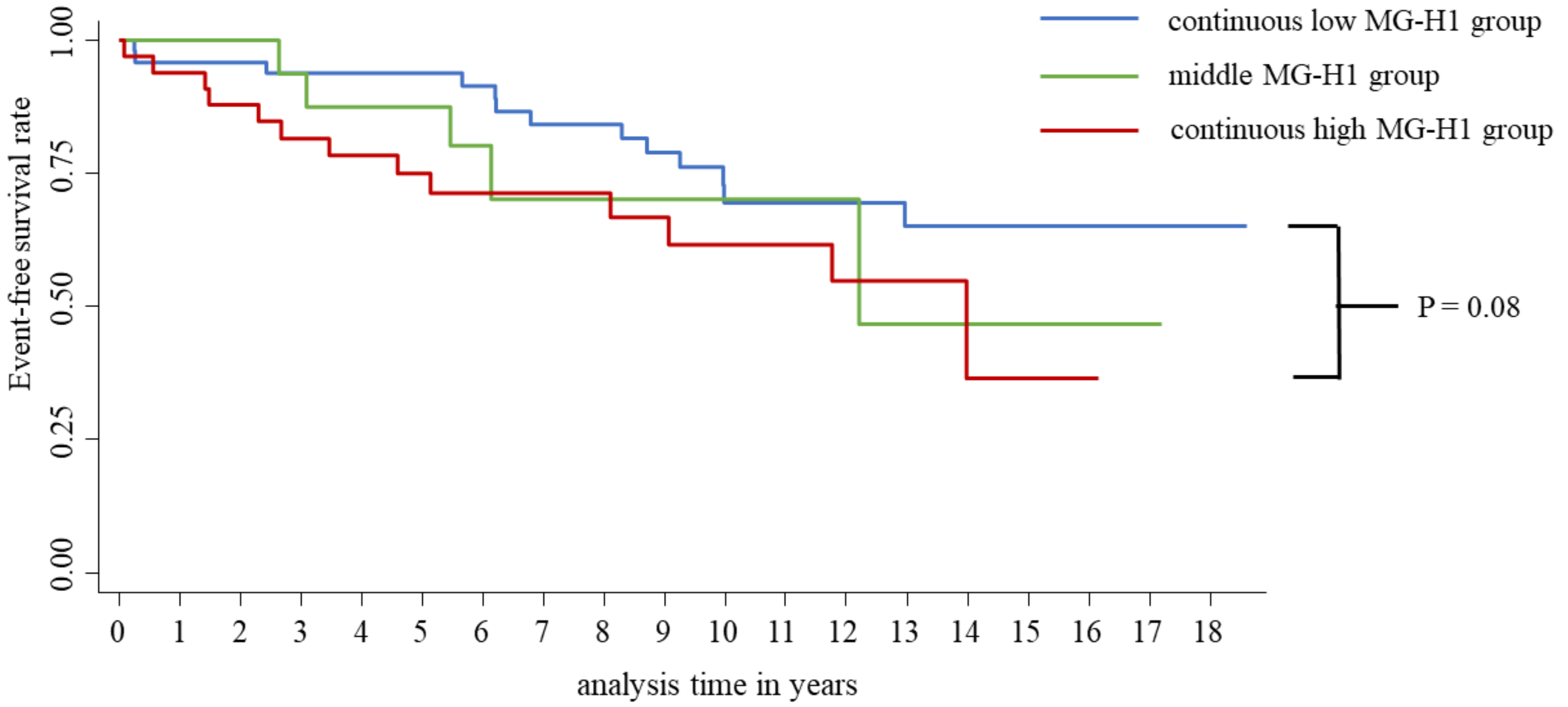

Supplement: Supplementary file 1 — Supplementary Material [file 12933_2023_1882_MOESM1_ESM.pdf]
